# Evaluation of a novel antibody to define histone 3.3 G34R mutant brain tumours

**DOI:** 10.1186/s40478-017-0449-1

**Published:** 2017-06-06

**Authors:** Farhana Haque, Pascale Varlet, Julien Puntonet, Lisa Storer, Aikaterini Bountali, Ruman Rahman, Jacques Grill, Angel M Carcaboso, Chris Jones, Robert Layfield, Richard G Grundy

**Affiliations:** 1 Children’s Brain Tumour Research Centre (CBTRC), School of Medicine, Queen’s Medical Centre, University of Nottingham, Nottingham, NG7 2UH UK; 2 Department of Neuropathology, Sainte-Anne Hospital, Paris, France; 3 Paris Descartes University, 1 rue Cabanis, 75674 cedex 14, Paris, France; 4 School of Life Sciences, Faculty of Natural Sciences, Keele University, Staffordshire, ST5 5BG UK; 5 Departement de Cancerologie de l’Enfant et de l’Adolescent et Unité Mixte de Recherche 8203 du Centre National de la Recherche Scientifique, Gustave Roussy et Universite Paris-Saclay, Villejuif, France; 6 Institut de Recerca Sant Joan de Deu, Barcelona, Spain; 7 Divisions of Molecular Pathology and Cancer Therapeutics, The Institute of Cancer Research, Sutton, Surrey SM2 5NG UK; 8 School of Life Sciences, Queen’s Medical Centre, University of Nottingham, Nottingham, NG7 2UH UK

**Keywords:** Histone mutations, H3.3, H3.1, DIPG, pHGG, Brain tumour

## Abstract

Missense somatic mutations affecting histone H3.1 and H3.3 proteins are now accepted as the hallmark of paediatric diffuse intrinsic pontine gliomas (DIPG), non-brain stem paediatric high grade gliomas (pHGG) as well as a subset of adult glioblastoma multiforme (GBM). Different mutations give rise to one of three amino acid substitutions at two critical positions within the histone tails, K27M, G34R/V. Several studies have highlighted gene expression and epigenetic changes associated with histone H3 mutations; however their precise roles in tumourigenesis remain incompletely understood. Determining how such amino acid substitutions in a protein affect its properties can be challenging because of difficulties in detecting and tracking mutant proteins within cells and tissues. Here we describe a strategy for the generation of antibodies to discriminate G34R and G34V mutant histone H3 proteins from their wild-type counterparts. Antibodies were validated by western blotting and immunocytochemistry, using recombinant H3.3 proteins and paediatric GBM cell lines. The H3-G34R antibody demonstrated a high degree of selectivity towards its target sequence. Accordingly, immunostaining on a cohort of 22 formalin-fixed paraffin embedded tumours with a previously known H3.3 G34R mutation status, detected successfully the corresponding mutant protein in 11/11 G34R cases. Since there was a high concordance between genotype and immunohistochemical analysis of G34R mutant tumour samples, we analysed a series of tissue microarrays (TMAs) to assess the specificity of the antibody in a range of paediatric brain tumours, and noted immunoreactivity in 2/634 cases. Importantly, we describe the generation and validation of highly specific antibodies for G34 mutations. Overall our work adds to an extremely valuable portfolio of antibodies, not only for histopathologic detection of tumour-associated mutant histone sequences, but also facilitating the study of spatial/anatomical aspects of tumour formation and the identification of downstream targets and pathways in malignant glioma progression.

## Introduction

Missense somatic mutations affecting histone H3.1 and H3.3 proteins are highly prevalent in diffuse midline gliomas and in a subset of hemispheric paediatric high-grade gliomas (pHGG) [5, 8, 9, 15], for which prognosis is very poor. The H3.3 mutation most commonly occurs in the *H3F3A* gene and is associated with one of three amino acid substitutions at two critical positions within the histone tails, which are K27M, G34R and G34V; whereas the H3.1 mutation mainly occurs in the *HIST1H3B* gene with K27M substitution [5, 8, 9, 15]. As mutations affecting regulatory genes are uncommon for diffuse intrinsic pontine gliomas (DIPG) and pHGG, this finding is extremely striking.

Distinct differences in the temporal, spatial and anatomical location of the histone H3 K27 and H3 G34 mutations have been noted; in particular, G34 mutations are found predominantly in supratentorial non midline tumours, whilst K27 mutations occur in more than 70% of DIPG as well as in mid brain tumours [5, 8, 9, 13, 15]. Importantly, long-term survivors of DIPG did not harbour K27 mutations in the H3.3 gene, with the K27 H3.3 mutation therefore defining a clinically and biologically distinct sub group of DIPG [8, 9, 13].

The development of effective therapies based on the underlying biology has been hampered by a lack of understanding of the molecular pathology of these tumours, in considerable part due to the prior scarcity of clinical tissue and patient-derived cell lines. The K27 and G34 mutations have been found to be associated with specific anatomical locations of distinct gene expression profiles and more recently with distinct epigenetic subgroups [3, 4, 12, 13]. For example H3.3 mutated tumours can be identified by differential protein expression patterns; K27 are OLIG2 positive and FOXG1 negative whilst G34 are OLIG2 negative and FOXG1 positive [13]. Recent developments have revealed that the H3 K27M mutation changes the epigenetic landscape by inhibiting the methyltransferase activity of EZH2 in the polycomb repressive complex 2 (PRC2), which leads to global reduction of K27me3 levels [11]. Despite these insights, the mechanistic roles of different histone mutations in gliomagenesis remain incompletely understood and have not yet led to the realisation of therapeutic targets [8].

To better understand the underlying biology of H3 mutations in brain tumours, different groups have applied a number of molecular approaches, including generation of a mutant selective antibody which recognises H3.1 and H3.3 K27M mutated residues [11]. Since then, various studies have utilised the H3-K27M antibody and have shown it to be effective by immunohistochemistry, where it demonstrated 100% sensitivity and specificity. Furthermore it has proved to be superior to a H3K27me3 antibody (which is used to screen global reduction of H3K27me3) in diagnosing H3 K27M mutations in brain tumours [2, 14]. In this study, we report the generation and application of novel antibodies against H3.3 G34R and G34V mutations. H3-G34R and H3-G34V antibodies were raised in rabbits and affinity purified. Both antibodies could detect exogenous and endogenous H3.3 G34R/V mutant proteins, by western blot and immunofluorescence methods. Importantly, the H3-G34R antibody effectively demonstrated high specificity and sensitivity to detect the G34R mutation in brain tumour sections by immunohistochemistry.

## Materials and methods

### Histone mutant specific antibody production

**Fig. 1:**
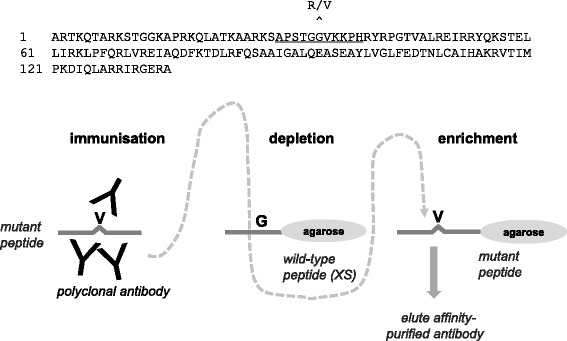
(*Top*) Human histone H3.3 protein sequence with underlined peptide sequence used for antibody production. The position of the H3 G34R/V mutations site is indicated. (*Below*) Schematic overview of the antibody generation protocol (H3-G34V antibody shown here) with depletion of crude antisera on wild-type peptide prior to affinity-enrichment on the antigenic peptide

Rabbit polyclonal antibodies against human histone H3.3 mutant proteins (G34R and G34V) were produced via a synthetic peptide approach with a commercial partner, Cambridge Research Biochemicals (Billingham, Cleveland). Peptide sequences that showed good antigenic probability were chosen for antibody production (Fig. 1). The strategy was to incorporate the mutated residue centrally within the peptide sequence, with five amino acids at either side. Conjugation of the synthetic peptide to a carrier prior to immunisation was achieved by the addition of an N-terminal Cys residue. The immunisation procedure was based on a 77-day schedule.

### Affinity purification of antibodies and ELISA

Crude antisera were first subjected to an initial immunodepletion step with an excess of the wild-type peptide sequence. A subsequent affinity-enrichment step was performed with antibodies by using their corresponding antigenic mutant peptides. For each rabbit the harvest bleed was passed by gravity over the affinity media in the column. The column was then washed with phosphate-buffered saline (PBS). The purified antibodies were eluted with two different pH buffers, firstly glycine buffer pH 2.5, followed by equilibration with PBS and a further elution with triethylamine (TEA) buffer pH 11. For the H3-G34R antibody, an additional depletion step using the wild-type peptide was also included as ELISA indicated residual cross-reactivity against this sequence after the initial depletion/purification step (data not shown). The eluates were then transferred to dialysis cassettes and dialysed against PBS, overnight with stirring at 4 °C. Antibody specificity was assessed by ELISA. 50 μl/ well of 10 μg/ml of the relevant peptide in 1× PBS buffer was used to coat an ELISA plate (Nuncproduct code 439454). The plate was incubated at 37 °C for 1 h. All the washing steps were with 1× TBS-Tween 20 (0.1% (*v*/v) Tween 20). Samples of crude unpurified antisera, unbound antisera from the purification column and the purified eluates were serially diluted from 1/50, 1/100, 1/200, 1/400, 1/800, 1/1600, 1/3200, 1/6400, 1/12,800, 1/25,600 and 1/51,200 in PBS-Tween 20 (0.1% (*v*/v) Tween 20), applied at 50 μl/well and incubated for 60 min at 37 °C. The reagent blank consisted of 50 μl PBS-Tween 20. Samples were removed and the wells washed. Polyclonal goat anti-rabbit immunoglobulin alkaline phosphatase secondary antibody (Vector product code AP-1000) was diluted 1/500 in PBS-Tween 20, applied at 50 μl/well and incubated for 60 min at 37 °C. 50 μl of the alkaline phosphatase substrate (Sigma product code N2770 p-Nitrophenyl Phosphate Tablets) was added and incubated for 30 min at 37 °C. The absorbance was determined at 405 nm on an Epoch Biotek plate reader. All sample values were corrected by subtracting against the reagent blank value, which was the average of 8 readings on every plate.

### Cell culture

Human paediatric glioblastoma (GBM) cell lines SF188 and KNS42 have been extensively characterised previously [1]. KNS42 harbours a histone H3.3 (*H3F3A*) G34V mutation whilst SF188 is H3 wild-type [4]. These cell lines were grown as monolayers in DMEM/F12 Ham’s medium (Life Technologies) supplemented with 10% fetal calf serum (GIBCO) and antibiotics at 37 °C and 5% CO_2_. Paediatric pHGG cell lines HSJD-DIPG-012 and HSJD-GBM-002 were grown according to the *in house* protocol from the Montero lab. Cells were cultured as monolayers in flasks pre-treated with laminin (10 μg/ml, BioLamina). Tumour Stem Medium (TSM) base was prepared with 50% Neurobasal-A Medium, 50% D-MEM/F-12, 1% HEPES buffer solution (1 M), 1% sodium pyruvate MEM Life 100MM(CE), 1% MEM non-essential amino acids solution 10 mM (100×), 1% GlutaMAX-I supplement and 1% antibiotic-antimycotic (100×) (LifeTechnologies). 10% FBS was added fresh to TSM base media for preparing a differentiation TSM media. Cells were grown as monolayers in this media at 37 °C with 5% CO_2_.

### Cloning, recombinant protein expression and western blotting

A plasmid construct pGEX-H3.3 for the expression of GST-tagged human histone H3.3 was generated by sub-cloning the H3.3 cDNA from pCDNA4/TO-FLAG-H3.3 (Addgene) into pGEX-4 T1 in frame with the *Eco*RΙ and *Bam*HΙ sites. pGEX-H3.3 was mutated at positions equivalent to G34R, G34V and K27M using mutant-specific primers in an overextended PCR method. All constructs were verified by DNA sequencing. GST-H3.3 protein expression in XL10 gold *E. coli* was induced by addition of 0.2 mM isopropyl-β-D-thiogalactopyranoside (IPTG) in the log phase of growth. PBS buffer supplemented with PMSF was used for re-suspension of the bacteria, which were lysed by sonication. Pull-downs were performed by binding GST fusion proteins to glutathione-Sepharose beads 4B (GE Healthcare). Sepharose-captured proteins were then probed with antibodies as follows. Protein were eluted from beads by boiling in an equal volume of 2 × Laemmli buffer, then separated by electrophoresis on 15% SDS-polyacrylamide gel, and transferred onto a nitrocellulose membrane. The primary antibodies used for probing were wild-type histone H3 (Abcam ab1791) (1/2500), H3-K27M (Milipore ABE419) (1/1000), and affinity-purified H3-G34V (1/500) and H3-G34R (1/250) antibodies (glycine elutes from this study) which were incubated either overnight at 4 °C or 1 h at room temperature. The primary antibodies were detected by horseradish peroxidase-conjugated anti-rabbit secondary antibodies (Dako) and visualized with the Western Lighting plus ECL detection system (Perkin Elmer). 5% (*w*/*v*) bovine serum albumin (BSA) in PBS was used for blocking the nitrocellulose membrane and 1% PBS-Tween was used for all washing steps.

### Immunofluorescence

KNS42, SF188, HSJD-DIPG-012 and HSJD-GBM-02 cells grown were grown on acid-treated glass coverslips and were fixed in methanol at −20 °C. Cells were washed in PBS, blocked with 1% (*w*/*v*) BSA in PBS and incubated with antibodies for wild-type histone H3 (Abcam ab1791), H3-K27M (Millipore ABE419) and affinity-purified H3-G34V and H3-G34R antibodies (glycine eluates from this study) diluting in 3% (*w*/*v*) BSA in PBS. Secondary antibodies were either goat anti-rabbit AlexaFluor 488 or goat anti-rabbit 568 (Invitrogen, Molecular Probes). Coverslips were mounted in Vectashield (Vector laboratories). Fluorescence microscopy was performed with a Deltavision microscope. Images were processed with Image J software.

### Tumour samples and Immunohistochemistry

The first study cohort comprised 22 formalin fixed paraffin embedded (FFPE) samples with a known histone genotype. These tumors were diagnosed between 2002 and 2015 as supratentorial high-grade glioma (HGGWHO III and IV: 14 grade IV glioblastomas, 3 grade III astrocytomas), 2 anaplastic gangliogliomas, 2 grade III oligo-astrocytomas, and 1 high grade glioma NOS. 11 HGG present with G34R H3F3A mutation by direct genomic sequencing and 11 did not (of the latter, 5 presented with K27M H3F3A mutation and 6 were “wild type” (WT), but showed an identical morphological and immunological phenotype with ATRX loss of expression and strong P53 nuclear immunopositivity). The second cohort comprised a panel of children’s TMAs containing a range of histopathological subtypes (83 medulloblastomas, 64 CNS PNETs, 91 Low grade Gliomas (LGGs) and 396 ependymomas) of childhood brain tumours, were also stained for immunohistochemistry (IHC) (Table 1).

Representative zinc formalin-fixed 4 μm sections or TMA were deparaffinized and were subjected to a Ventana autostainer (Discovery XT, Ventana Medical Systems, Tucson, USA). A standard pretreatment protocol included CC2 buffer and then affinity-purified rabbit polyclonal H3-G34R or H3-G34V antibodies (glycine elutes, diluted at 1:150 for G34R and 1:400 for G34V) incubation for 30 min at room temperature. Antibody binding was visualized with a ChromoMap detection kit (Ventana, Tucson, USA). Diaminobenzidine tetra hydrochloride (DAB, Ventana) was used as the chromogen. Slide scanning was performed using NanoZoomer 2.ORS (Hamamatsu photonics, Hamamatsu, Japan). Scoring was performed by a neuropathologist, blinded to the tumor genotype. In case of the TMA, the genotypes were not known. A sample was considered as positive for G34R if tumor cells showed a nuclear staining associated with the negativity of the control endothelial cell nuclei.

## Results

**Fig. 2:**
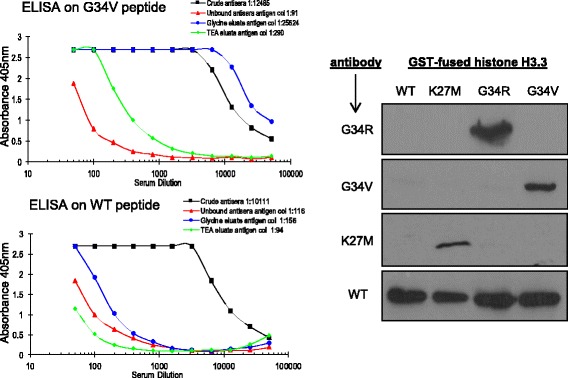
(*Left*) ELISA showing reactivity of crude antisera (*black*), unbound fraction after affinity enrichment step (*red*), and purified antibodies in glycine (*blue*) and TEA (*green*) elutions, against antigenic peptide (*top*, G34V) or the wild-type histone sequence (*below*). (Right) Western blot showing purified recombinant GST-histone proteins as indicated are selectively detected with different antibodies. H3-G34R (1/250) and H3-G34V (1/500) are antibodies generated in this study; H3-K27M (1/1000) and H3 wild-type (WT, 1/2000) are commercially available

Determining how a disease-associated amino acid substitution in a protein affects its properties can be challenging because of difficulties in detecting and tracking mutant proteins within cells/tissues. To directly address this problem for glioma-mutant histone sequences, we generated antibodies capable of selectively detecting mutant proteins over wild-type counterparts. Briefly polyclonal antibodies were raised in rabbits against synthetic histone peptide sequences (identical in H3.3 and H3.1) carrying G34R or G34V mutations, with an N-terminal Cys residue included to allow conjugation of synthetic peptide to carrier prior to immunisation (Fig. 1, top). Crude antisera were immunodepleted by passing through an excess of wild-type peptide immobilised on beads, then affinity-purified using the immobilised corresponding mutant peptides on beads, followed by final elution with either glycine or TEA buffer (Fig. 1, bottom). Specificity of purified antibodies was first confirmed by ELISA, against both the wild-type (depletion) and mutant (antigen) peptides. As an exemplar, data for the H3-G34V antibody is presented, demonstrating a strong preference of the glycine-eluted fraction for the mutant peptide (titre of 1:25,624 compared to titre of only 1:156 against the wild-type peptide) (Fig. 2, left).

Next we tested the ability of the purified (glycine-eluted) antibodies to detect the corresponding H3.3 mutant proteins. Whilst the work was in progress, a H3-K27M specific antibody was also reported [11] and commercialised and so was included in the analyses. Plasmids allowing expression of glutathione-S-Transferase tagged (GST)- H3.3 wild-type and mutant (G34R, G34V and K27M) proteins in bacteria were generated, and used to produce the histone sequences which were affinity-purified using glutathione-Sepharose chromatography. Western blotting against the GST-histone sequences using both our purified antibodies along with the commercial H3-K27M and anti-histone antibodies demonstrated selective detection of the respective mutant proteins, with no obvious cross-reactivity against the wild-type sequence (Fig. 2, right). However, upon longer incubation periods or at higher concentration the H3-G34V-selective antibody showed low cross-reactivity against the G34R protein (but not against K27M or wild-type, data not shown).

**Fig. 3:**
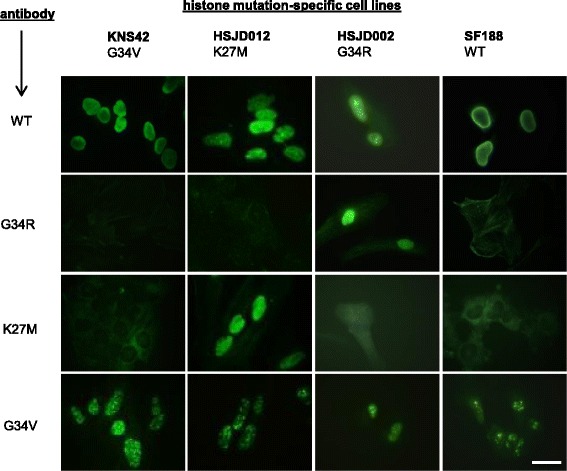
Patient-derived cell lines with indicated histone mutations stained with different antibodies (all 1:100) and detected by immunofluorescence microscopy (H3-G34R and H3-G34V antibodies generated in this study; H3-K27M and H3 wild-type (WT) antibodies are commercially available). (Scale bar 15 μm)

To further probe the specificity of the antibodies, we tested if they could detect endogenously expressed mutant H3.3 proteins. Four cell lines were cultured as representative models; SF188 (negative control, wild-type histone), KNS42 (H3.3-G34V), HSJD-DIPG-012 (H3.3-K27M) and HSJD-GBM-002 (H3.3-G34R). Antibodies were used to stain cultured cells grown in differentiating TSM media on cover-slips and visualised by immunofluorescence microscopy (Fig 3). Consistent with the low cross-reactivity noted by western blotting (see above), our H3-G34 V antibody showed weak nuclear staining of not only the KNS42 (G34V) cells but also of SF188 (wild-type), HSJD-DIPG-012 (K27M) and HSJD-GBM-002 (G34R) cells. Further purification of the H3-G34V antibody may enhance its usability for this application. However, the H3-G34R antibody demonstrated the desired specificity, showing nuclear staining only of the HSJD-GBM-002 (G34R) cells. Consequently, the H3-G34R antibody was taken forward for further validation for immunohistochemistry using surgically resected tissues.

**Fig. 4:**
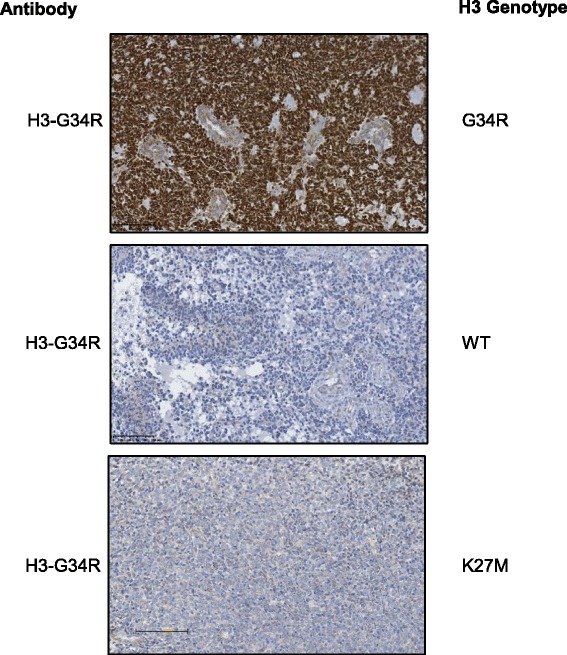
H3-G34R antibody immunostaining correlates with histone H3.3 genotyping. Representative IHC using H3-G34R antibody on a H3 G34R mutated tumor section shows a strong nuclear positivity in nearly all tumor cells but no staining in the nuclei of endothelial cells (*top*). WT H3 tumor is negative with H3-G34R antibody by IHC (*middle*). H3 K27M thalamic tumor stained negative for H3-G34R antibody by IHC (*bottom*). (scale bar 100 μm)

Indeed staining tumour sections from a cohort of high-grade gliomas demonstrated the specificity of our H3-G34R antibody. Twenty-two tumour FFPE samples with known H3 genotype (diagnosed as supratentorial high-grade glioma, glioblastomas, astrocytomas, anaplastic gangliogliomas, oligo-astrocytomas and high grade glioma) were stained. Out of these samples 11 HGG had G34R mutation, 5 had K27M mutation and 6 were H3 WT. The H3-G34R antibody successfully detected the corresponding endogenous H3 G34R mutant protein by immunohistochemistry in all (11/11) G34R mutated tumors. The antibody showed a strong nuclear staining in majority of tumor cells (> 90% of tumor nuclei). Endothelial and normal residual glial and neuronal cells were not immunostained. One representative stained section shown in Fig. 4 (top), and importantly, none of the H3.3 G34 WT (*n* = 6) or K27M (*n* = 5) mutant tumors showed nuclear staining with the H3-G34R antibody (Fig. 4, middle, bottom).

Having established that the H3-G34R antibody detected the correct mutation in HGG, we assessed the specificity of the antibody in identification of G34R mutation against a range of different histological subtypes of childhood brain tumours, which include tumours previously diagnosed as CNS PNET, medulloblastoma, low grade glioma and ependymoma. We evaluated staining of CBTRC (Children’s Brain Tumour Research Centre) TMAs representing above histopathological subtypes of childhood brain tumours, and found that where only 2 out of 634 cases were G34R positive. The positive cases were respectively a low grade glioma and a CNS PNET (Tables 1 and 2). This demonstrates that the H3-G34R antibody does not have high cross reactivity and is specific, which can be used to rapidly detect H3.3 G34R mutants in an extensive collection of tumour tissues.

**Table 1:** H3-G34R antibody was tested by immunohistochemistry against a panel of children’s tissue micro-array containing a range of childhood brain tumours

Diagnosis	No. of Cases stained	G34R positive
Medulloblastoma	83	0
CNS PNET	64	1
Low Grade Glioma	91	1
Ependymoma	396	0
Total	634	2

**Table 2:** Clinical information on positive G34R tissue micro-array cases of childhood brain tumours

Case	Age at diagnosis (yrs)	Sex	Location
LGG 1	12.5	F	Posterior Fossa
CNSs PNET	15.3	F	Temporal/hippocampus

## Discussion

Childhood brain tumours are the major cause of cancer related death in childhood. Highest amongst these are histone 3 mutation-associated brain tumours, DIPG and pHGG, which have dismal prognoses. Histone mutations in brain tumours occur mainly at the K27 and G34 position in the *H3F3A* gene (80% of DIPG and 30% of pHGG), and less commonly at the K27 position in the *HIST1H3B* gene [8, 9, 15]. The K27M mutation has been shown to inhibit the methyltransferase activity of EZH2 in the PRC2 complex that leads to global reduction of H3K27me3 levels [11]. In contrast, it is speculated that the G34V/R mutations affect global H3K36 trimethylation by inhibiting trimethyltransferase SETD2 [8]. Although there have been substantial advances in the molecular understanding of histone mutant brain tumours, we still lack rapid diagnostic tools and effective therapeutic targets to benefit these paediatric patient groups.

To expand our understanding of these tumours, we have developed new polyclonal antibodies against H3-G34R/V mutants. In order to obtain these mutant-specific antibodies, we used a small peptide (11 residues) containing the appropriate H3.3 mutation as an antigen, to induce immunogenicity in rabbits. These antibodies were then affinity purified to further enhance their specificity. A H3-K27M antibody has been already reported and been commercialised by a different group [11]. Here, we have reported the analyses and usability of our *in house* generated H3-G34R and H3-G34V antibodies.

Both H3-G34R and H3-G34V antibodies displayed high titre in detecting the corresponding mutant antigens by ELISA, consistent with the specific detection of both exogenous and endogenous H3 G34R/V mutant proteins, by western blot and immunofluorescence. The H3-G34R antibody is now ready for further applications, such as protein interaction and pathway analysis, to better elucidate the pathology of pHGG. However, the H3-G34V antibody exhibited cross reactivity with the G34R mutant recombinant protein by western blot and displayed slight background in the nucleus by immunofluorescence experiments. Nonetheless, from our results the H3-G34R antibody is anticipated to have more selective and wide applicability in the DIPG/pHGG research fields.

We next assessed the effectiveness of these antibodies by immunohistochemical methods to diagnose the H3 G34R/V mutation associated brain tumours. The H3-K27M antibody has previously been shown to be specific and sensitive in detecting the H3 K27M mutation in brain tumour sections [2, 6, 14]. In our study, we have now successfully demonstrated that the H3-G34R antibody is also highly selective and sensitive in detecting H3-G34R mutation in brain tumour sections and TMAs. However, G34R antibody staining of TMAs revealed that one LGG case out of 91 showed positive staining for G34R. As this child is now deceased, we could not verify whether it was a possible misdiagnosis or not. Unfortunately further tissue was not available for histological review or sequencing. The other G34R positive sample was previously classified as a CNS PNET based on histological and previously accepted immunohistochemical markers. This represents one out of 64 cases, and may reflect the known challenges of diagnosis high grade supratentorial CNS malignancies in childhood which can now be facilitated by specific antibodies such as reported here in the light of recent proposal for reclassification of CNS PNETs [10]. Interestingly, Gessi et al. have recently reported G34R mutations in 4/33 CNS-PNET cases by sequencing and suggested that these could be defined tumour subtype [7], supporting the potential of our G34R antibody to detect G34R mutation in various TMA cohorts. However, once again further tissue was also not available for our CNS-PNET case to histological review or sequence. It is unfortunate that we were unable to genotype the two tumours, in both cases the biopsy samples were very small and the tissues predominantly used for the diagnostic process. This limitation in part provided our motivation for generating new diagnostic tools. In the future access to a larger cohort of H3.3 mutant tumours will allow us to confirm the full specificity of our antibody. Considering all of the above, our novel G34R antibody demonstrates its utility as an effective diagnostic tool for rapid detection of the histone 3 mutational statuses of brain tumours. Additionally, the H3-G34R antibody was validated and confirmed to be suitable for chromatin immunoprecipitation (ChIP) analyses, by Active Motif (Carlsbad, CA, USA) (data not shown). The ChIP grade quality of our G34R antibody is valuable for future investigations to probe the mechanism of these H3 mutant brain tumours.

## Conclusions

To date no biomarker or diagnostic tool to detect H3 G34R/V mutant tumours is available for the clinical or laboratory setting. The H3-G34R/V antibodies described here have the potential to be effective in this regard. Our G34R antibody is highly selective, and now ready for use in further research in patient diagnostic purposes. Furthermore, these antibodies will provide a valuable tool to promote our understanding of the molecular biology of H3 G34R/V mutant brain tumours in comparison with the H3 K27M mutant tumours. However, only a large-scale evaluation of these antibodies can reveal their true benefit.

## Abbreviations

CBTRC: Children’s Brain Tumour Research Centre
ChIP: Chromatin immunoprecipitation
DIPG: Diffuse intrinsic pontine gliomas
FFPE: Formalin fixed paraffin embedded
GBM: Glioblastoma multiforme
IHC: Immunohistochemistry
LGG: Low grade Glioma
pHGG: Paediatric high grade glioma
PRC2: Polycomb repressive complex 2
TMAs: Tissue microarrays
TSM: Tumour Stem Medium
WT: Wild Type
